# A Desirable Kind of Contract Practice

**Published:** 1913-11

**Authors:** 


### A Desirable Kind of Contract Practice

A church of six hundred members, including all ages, has decided to employ an assistant minister. Its present minister is paid a salary about equal to the earnings of the average successful physician supported by a clientele of the same average social status, but, unlike the physician, receives many perquisites and a two months' vacation every year. The assistant is to be a young man and is to receive a beginner's wage of $1200. (We earned about $500 the first year, collected half of it and earned about as much more quizzing students. One of our friends was lucky enough to get a position as assistant to an old physician at $8 per week.) It is estimated that the assistant, by his parochial work will add to the congregation sufficiently to make up the extra expense.

This form of liberality, or let us say, recognition of the value of spiritual services, is liable to cause the envy of the physician, who, with the same average support of 600 persons, has a hard row to hoe. Personally, we are glad to witness such evidence of appreciation and of determination to supplement the labors of the spiritual leader. But why would not something of the same practical spirit, not necessarily carried out in exactly the same way, be applicable to the one who cares for the bodies—and, incidentally, may have about as good an influence on the moral and religious health of the community?

